# Evapotranspiration dynamics and their drivers in a temperate mixed forest in northeast China

**DOI:** 10.7717/peerj.13549

**Published:** 2022-06-08

**Authors:** Xiaoying Wang, Xianjin Zhu, Mingjie Xu, RiHong Wen, Qingyu Jia, YanBing Xie, Hongda Ma

**Affiliations:** 1 Institute of Atmospheric Environment, China Meteorological Administration, Shenyang, People’s Republic of China; 2 Shenyang Agricultural University, College of Agronomy, Shenyang, People’s Republic of China; 3 Yichun Wuying District Meteorological Service, Yichun, People’s Republic of China

**Keywords:** Water cycle, Evaptranspiration, Forest, Cool temperate, Eddy covariance, Terrestrial ecosystem, Climate change

## Abstract

Evapotranspiration (ET) is a vital part of the global water cycle and is closely related to carbon sequestration. Analysing ET dynamics and their drivers would benefit for improving our understanding of the global water and carbon cycles. Using an eddy covariance (EC) approach, we analysed ET dynamics and their drivers in a temperate mixed forest over northeast China from 2016 to 2017. The results showed that 43.55% of our eddy covariance data passed the quality control. In addition, the energy balance ratio was 0.62, indicating that measurements were reliable. The measured ET showed clear single peak patterns with seasonal and diurnal variations. The daily ET ranged from 0 to 7.75 mm d^−1^ and the hourly ET ranged from 0 to 0.28 mm h^−1^. The ranges of hourly ET floated from 0 to 0.05 mm h^−1^ at non-growing season (November to April) while ranged from 0 to 0.28 mm h^−1^ at active growing season (May to October). The diurnal ET dynamics during the non-growing season were driven by air temperature (*T*_a_)_,_ but were governed by global radiation (*R*_g_) during the active growing season. Leaf area index (LAI) comprehensively reflected the variations of *T*_a_ and *R*_g_, and was found to be the primary factor shaping the seasonal dynamics of ET. The annual ET rates were 501.91 ± 5.30 mm year^−1^ and 554.60 ± 11.24 mm year^−1^ for 2016 and 2017, respectively. Therefore, energy supply, represented by *T*_a_ and *R*_g_, governed ET dynamics in our temperate mixed forest, while variables representing the energy supply affecting ET dynamics differed among seasons and time scales. ET dynamics indicated that a temperate mixed forest is important to the global water cycle. Our results improved our understanding of ET dynamics in the studied region.

## Introduction

Evapotranspiration (ET) is defined as the water lost as vapour rising from the land to the atmosphere. It is a vital part of the global water cycle (Kool et al., 2014; Wang et al., 2010; Wang & Dickinson, 2012). ET is also closely associated with carbon sequestration as water loss and carbon sequestration both primarily appear in the stoma of a plant (Chapin, Matson & Mooney, 2012), which made ET closely associate with the global carbon cycle (Zhu et al., 2015b). Analysing the dynamics of ET and their drivers is beneficial for understanding the mechanisms underlying ET dynamics, thus accurately modeling ET. This also results in a better understanding of the global water and carbon cycles (Anapalli et al., 2020).

Analysing ET dynamics and their drivers requires accurate measurements (Li et al., 2009; Rana & Katerji, 2000; Wang & Dickinson, 2012). The micrometeorological theory known as eddy covariance (EC) has been widely used in measuring ET for its *in-situ*, continuous, and accurate measurements (Baldocchi & Ryu, 2011; Drexler et al., 2004; Tanaka et al., 2008). Many studies have analysed ET dynamics and their drivers for various ecosystems using EC (Cristiano et al., 2015; Kume et al., 2011; Ma et al., 2015; Song et al., 2017; Xu et al., 2014; Yue et al., 2019). Results showed that daily accumulated ET and their annual values differed among ecosystems (Kumagai et al., 2005; Li et al., 2010; Tong et al., 2017). Some studies have analysed ET dynamics and their drivers for temperate forests in North America (Schaefer et al., 2014; Wang et al., 2015; Wehr et al., 2017), Europe (Gielen et al., 2010; Pita et al., 2013; Soubie et al., 2016), and Asia (Tsuruta et al., 2016; Wu et al., 2013; Zhang et al., 2012). Carbon fluxes (Ohtsuka et al., 2005; Saigusa et al., 2002) and resource use efficiency (Okada, Takagi & Nishida, 2019) in temperate mixed forests over Asia have also been intensively reported, while less attention has been given to ET dynamics and what drives them in these ecosystems, which made the ET dynamics in this region poorly understood. In addition, temperate mixed forests over Asia are climax communities and the succession climax indicates the potential of this region to regulate regional energy and mass cycles (Zhang, Han & Yu, 2006; Zhang et al., 2006b). Analysing ET dynamics and their drivers in temperate mixed forests will improve our understanding of ET dynamics in this region, which benefited for revealing the potential role of this region in global water cycles.

We analysed ET dynamics and their drivers for a temperate mixed forest based on EC measurements in a broad-leaved Korean pine forest in northeast China. The main objectives of this study were: (1) to reveal ET dynamics for a temperate mixed forest in Northeast China, (2) to clarify the major drivers of ET dynamics, and (3) to reveal the magnitude of annual ET in this unique ecosystem. In order to address these objectives, we first addressed the energy balance closure of the eddy covariance measurements, which was directly related to the ET measurements (Wilson et al., 2002). We then showed the dynamics of ET and their environmental factors, including solar radiation (*R*_g_), air temperature (*T*_a_), relative humidity (RH), precipitation, and leaf area index (LAI) to determine the drivers of ET dynamics. Then the drivers of ET dynamics were performed. Our results elucidate the understanding of ET dynamics and ET modeling over a temperate mixed forest, which improves our understanding of the water cycle processes and their linked carbon cycles.

## Materials and Methods

### Site description

**Figure 1:**
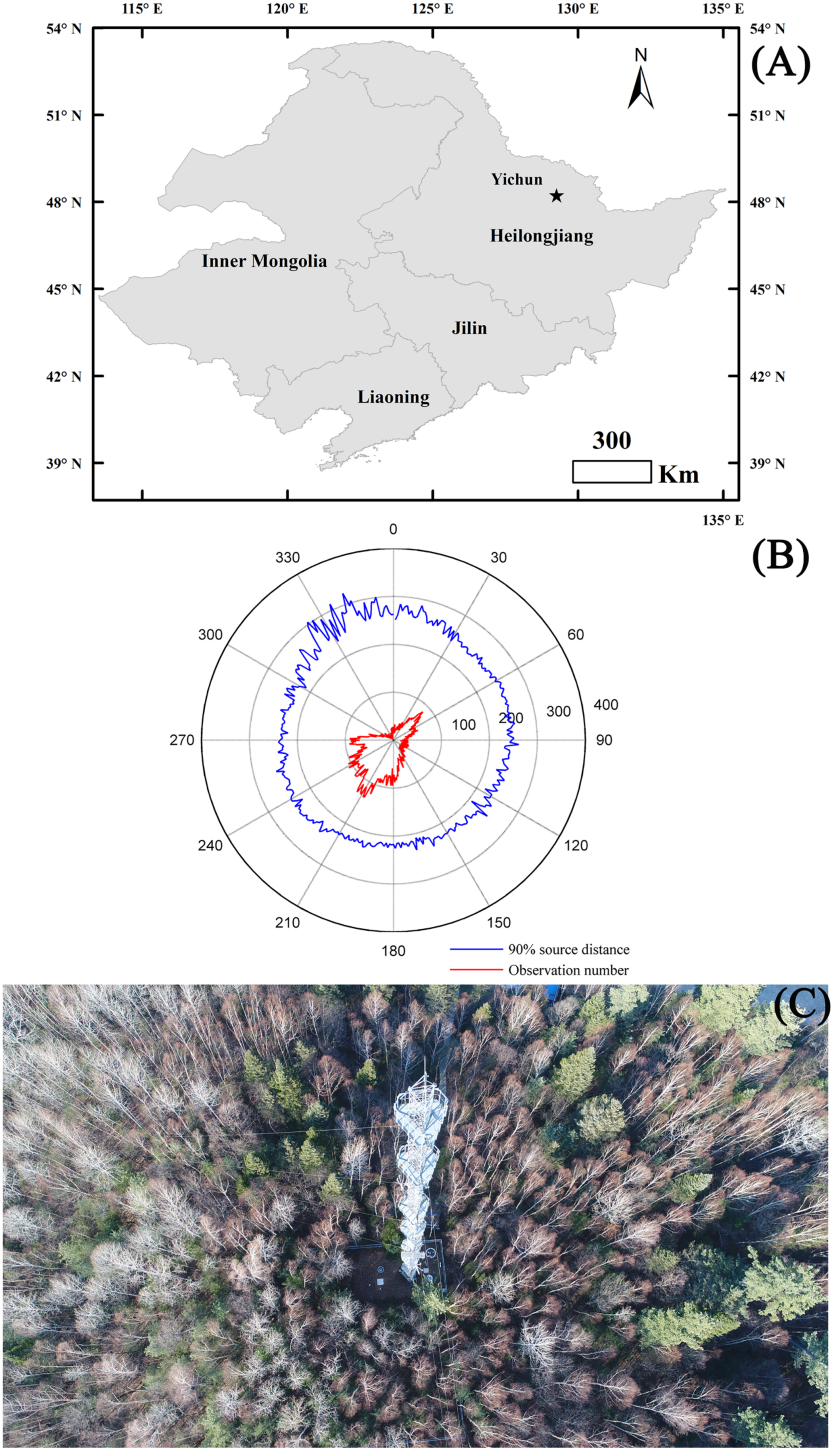
Site information. The ecosystem location (A) and its main characteristics, including the main wind directions and footprint (B), and the main plant composition (C). The footprint was calculated following Kljun et al. (2004) after calculating the mean of each degree. The maps of panel (A) were made by ArcGIS 10.0 (http://www.esri.com/software/arcgis).

EC measurements were taken at the Yichun broad-leaved Korean pine forest experimental station (48.2292°N, 129.2661°E, 342 m.a.s.l) from January 2016 to December 2017. This station was located in the middle of Heilongjiang Province, which was the northeastern-most province of China (Fig. 1A). The experimental ecosystem had a flat topography with an evenly distributed mixed forest (14,141 hm^−2^). The mean fetch of the tower in all directions was lower than 400 m, with the prevailing southwest and northeast wind directions (Fig. 1B). The forest was primarily comprised of Korean Pine (*Pinus koraiensis*), Mongolian Oak (*Quercus mongolica)*, Amur Linden (*Tilia amurensis*), and Maple Birch (*Betula costata*), with a density of 1,152 plants per hm^−2^ and an average stand age of 220-years (Fig. 1C). Korean Pine (*Pinus koraiensis*), Mongolian Oak (*Quercus mongolica)*, Amur Linden (*Tilia amurensis*), and Maple Birch (*Betula costata*) accounted for 10–15% of all plants in the study area, respectively. The mean canopy height was 26 m with a maximum and minimum LAI of 6.2 and 0.5 m^2^ m^−2^, respectively. The experimental forest had a temperate continental climate with a long-term mean annual air temperature (MAT) of 0.63 °C and a mean annual precipitation (MAP) of 610.7 mm. The highest *T*_a_ appeared in July with a long-term mean (1981–2010) value of 20.4 °C, while the lowest *T*_a_ occurred in January with a value of −22.5 °C. The greatest amount of precipitation occurred in July (147 mm), while the lowest precipitation occurred in February (6.7 mm). Precipitation was commonly seen as snowfall from November to April, and totaled approximately 90 mm year^−1^. The soil in the experimental ecosystem was characterized as dark brown soil with high soil organic matter content.

### Data measurements

We employed a flux tower with a height of 70 m to measure the ET and conventional meteorological variable dynamics.

The EC system was composed of a datalogger (Model CR5000; Campbell Scientific Inc., Logan, UT, USA), a 3-D sonic anemometer (Model CSAT3; Campbell Scientific Inc., Logan, UT, USA), and an infrared gas analyzer (Model LI-7500; Licor Inc., Lincoln, NE, USA). These were used to measure the net H_2_O exchange between the terrestrial ecosystem and the atmosphere, which can be considered as the ET. The EC sensors, including CSAT3 and LI-7500, were mounted at a height of 50 m, which was about twice of the canopy height. The EC measurement sampled the raw data with a frequency of 20 Hz and recorded the flux data at 30-min intervals.

Conventional meteorological variables were also measured, including air temperature (*T*_a_), relative humidity (RH), soil temperature (*T*_s_), precipitation, and soil heat flux (*G*). *T*_a_ and RH were measured at different heights (1.5, 20, 30, 40, 50, 60 m) with shielded and aspirated probes (Model HMP45C; Campbell Scientific Inc., Logan, UT, USA). *T*_s_ was measured using thermocouple probes (Model 105T; Campbell Scientific Inc., Logan, UT, USA) at different depths (0, 5, 10, 15, 20, 40, 80, 160, 320 cm). Precipitation was recorded with a rain gauge (Model 52203; R.M. Young Company, Traverse City, MI, USA) at the height of 38 m. *G* was measured with two flux plates (Model HFP01SC; Campbell Scientific Inc., Logan, UT, USA) at depths of 5 and 15 cm, respectively. Conventional meteorological variables were measured at a frequency of 1 s and recorded at 30-min intervals with dataloggers (Model CR10X & CR23X; Campbell Scientific Inc., Logan, UT, USA).

Radiation data, including global radiation (*R*_g_), reflected radiation (*R*_r_), downward long wave radiation (DLR), and upward long wave radiation (ULR), were measured with a radiation heat balance station (CAMS620-SP, Huatron, China) at 1-h intervals. The measured 1-h data were linearly interpolated into half-hour scale to match the time series of ET and other environmental variables. Net radiation (*R*_n_) was calculated as the sum of net short-wave radiation (*R*_ns_) and net long-wave radiation (*R*_nl_), where *R*_ns_ was the difference between *R*_g_ and *R*_r_ while *R*_nl_ was the difference between DLR and ULR.

### Data processing

#### Flux data processing

The net H_2_O exchange, which was deemed as ET and latent heat (*LE*), was calculated as the covariance between vertical wind speed (*v*) fluctuations and water vapor concentration fluctuations. The spikes of wind speeds and H_2_O concentrations were detected and deleted based on CSAT3 measuring three-dimension wind speeds and LI-7500 measuring H_2_O concentrations (Vickers & Mahrt, 1997). The raw ET was deemed as missing if the missing or deleted data were more than 3,600 in each half-hour (10% of all measurements) (Wen et al., 2010). The raw ET was calculated as the covariance of vertical wind (*v*) speed fluctuations and H_2_O concentration fluctuations using the despiked data. The calculated raw fluxes were derived from a three-dimensional rotation (Aubinet et al., 2000), Webb, Pearman and Leuning (WPL) correction (Webb, Pearman & Leuning, 1980), spectral correction for high-frequency losses (Moncrieff et al., 1997), low-frequency losses (Moncrieff et al., 2005), physical instrument separation losses (Horst & Lenschow, 2009), storage calculation (Hollinger et al., 1994), and footprint analysis (Kljun et al., 2004) calculated using online Eddypro 4.2 software (Licor Inc., Lincoln, NE, USA). The Eddypro proceeding water vapor fluxes were flagged with different numbers, where 0 and 1 indicated high quality fluxes that were suitable for ET dynamics analysis, while 2 indicated poor quality fluxes that should be discarded. The Eddypro data for ET flagged as 0 and 1 were further filtered for precipitation, threshold deleting, and low turbulence. The ET fluxes selected during a precipitation event and the following half hour were deleted as the sensors may suffer from moisture contact. The selected ET lower than −0.32 mm h^−1^ or higher than 1.30 mm per hour were deleted. Low turbulence was determined by the u* threshold, which was calculated following Reichstein et al. (2005) using u*, CO_2_ fluxes, and *T*_a_. The calculated u* thresholds were 0.25 and 0.21 m s^−1^ for 2016 and 2017, respectively. The data coverage after data quality control was 42.55% and 44.55% for 2016 and 2017, respectively. However, the data coverage showed obvious differences between daytime and nighttime. During the daytime, the data coverage was 66.66% and 66.28% for 2016 and 2017, respectively. However, nighttime only had 18.48% and 22.82% data coverage for 2016 and 2017, respectively. This was primarily affected by the low turbulence at night.

The ET data gaps were filled using the look-up table method (Falge et al., 2001a; Reichstein et al., 2005) based on *T*_a_, vapor pressure deficit (VPD), and *R*_n_.

CO_2_ fluxes were measured simultaneously using the same EC system and were calculated as the covariance between vertical wind speed (*v*) fluctuations and CO_2_ concentration fluctuations. Heat fluxes (*H*) were calculated as the covariance between vertical wind speed (*v*) fluctuations and air temperature fluctuations. The raw H fluxes suffered from quality control issues similar to those of ET.

*G* was also subjected to quality control by threshold values. Data gaps for *G* were linearly interpolated for gaps of less than 2 h but were filled using the mean diurnal variation (MDV) method for longer periods (Falge et al., 2001b).

#### Auxiliary data processing

The auxiliary data including conventional meteorological data and biotic data were also subjected to data quality controls and gap-fillings.

*T*_a_ and RH above the canopy (at the height of 30 m) were used to analyze the drivers of ET dynamics. The qualities of *T*_a_ and RH were firstly controlled through threshold values. The measured *T*_a_ lower than −50 °C or higher than 50 °C were deleted, while the threshold values of RH were 0% and 100%. In addition, the measured *T*_a_ and RH lower than 70% or higher than 130% of the mean corresponding values of other layers were deleted. The data gaps of meteorological variables (including *T*_a_ and RH) were filled using the linear interpolation for gaps of less than 2 h. The remaining data gaps were firstly filled with the corresponding variables in neighboring layers. Then the remaining data gaps were filled with the mean diurnal variation (MDV) method (Falge et al., 2001b). VPD was then calculated as the difference between the actual and saturation vapor pressures based on the measured RH and *T*_a_.

Precipitation data were quality checked and any gaps were filled using manual observations from a meteorological station affiliated with the Chinese Bureau of Meteorology, which was located approximately 18 km away from the observation tower.

*R*_g_ was quality controlled with spikes deletion. *R*_g_ data lower than 0 or higher than 1,300 W m^−2^ were removed. The data gaps of *R*_g_ were filled using the linear interpolation for gaps of less than 2 h, while the remaining data gaps were filled with the MDV method (Falge et al., 2001b).

*R*_n_ was also quality checked with spikes deletion used to estimate the energy balance closure. First, short wave radiation data lower than 0 or higher than 1,300 W m^−2^ were removed. Second, long wave radiation data lower than 100 W m^−2^ or higher than 600 W m^−2^ were removed. Only data passing the quality control were used to calculate *R*_n_.

LAI was downloaded from the Moderate Resolution Imaging Spectroradiometer (MODIS) database with a spatial resolution of 500 m and a temporal variation of 4 days (https://modis.ornl.gov/subsetdata). In addition, the pixels from the surrounding 1 km area were also downloaded to avoid potential noises in a single pixel, which all had the same mixed forest. The downloaded LAI were linearly interpolated into a daily scale.

### Data analysis

#### Energy balance closure assessment

We quantified the energy balance closure using two methods to validate the performance of the eddy covariance measurements. The first method derived the linear regression between the half-hour dependent flux variables *(LE* + *H)* and the available energy (*R*_n_ − *G*) from the ordinary least squares (OLSs) relationship (Li et al., 2005). The regression coefficients, including the slope and the intercept, were used to evaluate the performance of energy balance closure. The ideal closure was reflected by a one slope and a zero intercept. The second method derived the cumulative sum of *LE* + *H* and available energy (*R*_n_ − *G*) over measuring period and calculated the energy balance ratio (EBR) as follows:

(1) $${\rm EBR} = {{\rm sum}{(LE+H)}/{\rm sum}{(R_n-G)}}$$

Only data directly measured and passing the quality control were used to calculate the energy balance closure. All available data were first potted to generate the energy balance closure during the measuring period. Then the whole year was divided into the active growing season (May-October) and the non-growing season (November to April), to generate the energy balance closure at different seasons.

#### ET dynamics and their drivers

Daily ET and environmental variables of each year were described to illustrate their seasonal dynamics. These were then used to reveal the drivers of the seasonal dynamics of ET. Only the data measured above the canopy (30 m) was used, although multilayer observations of *T*_a_, RH, and VPD were obtained. To avoid the confusion of gap-filling data in representing ET seasonal dynamics and their drivers, we only used daily data during days having more than 20 (including 20) directly measured and passing the quality control data. Setting 20 as the minimum data number sourced from the following four aspects. First, the missing of ET primarily resulting from low turbulence during nighttime made no day have all half-hour data directly measured. Second, data gaps were primarily caused by ET missing as nearly all *R*_g_ were directly measured when the number of directly measured ET were over 20. Third, the small ET values during nighttime made the uncertainties in gap-filling contribute little to the daily accumulated values. Fourth, the occasionally appearing data gaps during daytime could be linear interpolated with high confidence.

The data from an entire year were divided into two groups: active growing season and non-growing season. The active growing season lasted from May to October and the non-growing season included the remaining months. We calculated the mean ET for each group over each half-hour and their corresponding variables, including *T*_a_, *R*_g_, and VPD, to represent the diurnal variations in different seasons. Only data directly measured and passing the quality control were used. Each available half-hour data point in each group was used to analyse the drivers of the diurnal variations of ET. Each year data were used seperately to validate the results from each year.

Though *R*_n_ was the direct variable reflecting the available energy, the calculation of *R*_n_ may be inaccurate as the bad sensors especially the long wave radiation sensors. Therefore, *R*_g_ was used to replace *R*_n_ to reveal the drivers of ET dynamics.

### Uncertainty analysis

A simple Monte Carlo experiment was conducted to assess the uncertainty in the annual estimates of ET following Desai et al. (2005). A random number generator was used to remove 10–50% of the existing observed data. The generated data gaps were then randomly selected and filled with modeled ET (described in Flux processing), which was run 100 times for each year (2016 and 2017).

### Statistical analysis

MATLAB 2014a (Mathworks Inc., Natick, MA, USA) was used to process the data including the flux quality control and gap filling. Linear regression and non-linear regression were used to analyse the effects of various factors on dynamics (including the diurnal and seasonal dynamics) of ET. Stepwise regression was used to analyze the multiple linear regression on ET seasonal variations. The minimum P-value for a variable to be added or removed from the model in the stepwise regression was 0.10. A path-analysis was conducted to evaluate the dependence of ET on various factors. The significant level was set to *α* = 0.05.

## Results

### The energy balance closure

**Figure 2:**
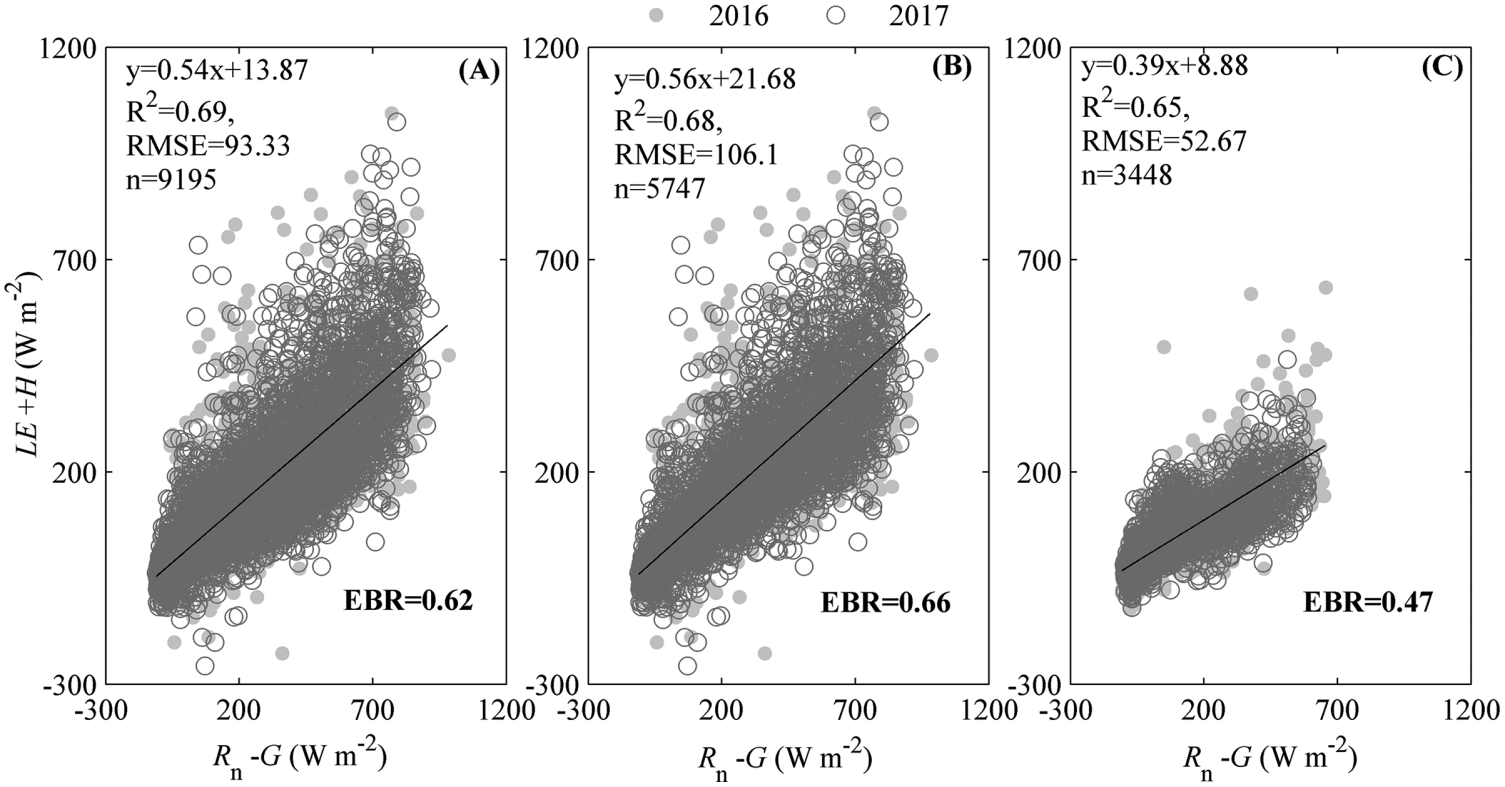
The energy balance closure calculated by the ordinary least squares (OLSs) relationship and energy balance ratio (EBR). The OLSs relationship was calculated between the available energy (*R*_n_–*G*) and the energy fluxes in the studied ecosystem during measuring period. The available energy was the difference between the net radiation (*R*_n_) and soil heat flux (*G*). The energy fluxes were the sum of heat fluxes (*H*) and latent heat fluxes (*LE*). EBR was calculated as the ratio of total energy fluxes to available energy during the measuring period, was also calculated. Only data passing quality control were used. The energy balance closure was calculated during the whole measuring period (A), the active growing season (B), and non-growing season (C).

**Figure 3:**
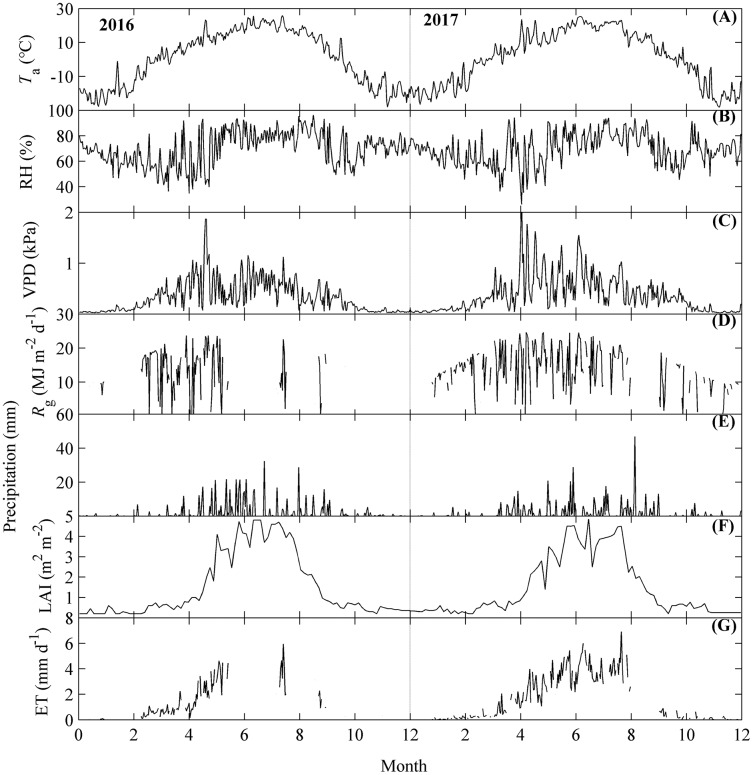
The seasonal dynamics of environmental factors and evapotranspiration (ET). Environmental factors included air temperature (*T*_a_, A), relative humidity (RH, B), vapor pressure deficit (VPD, C), global radiation (*R*_g_, D), precipitation (E), and leaf area index (LAI, F). Seasonal variations of *T*_a_, RH, VPD, and LAI were calculated as their daily mean values, while those of *R*_g_, precipitation and ET (G) were calculated by their daily accumulated values. Only daily data during days having more than 20 (including 20) directly measured and passing the quality control data were used.

The energy balance closure indicated by the regression slope between the available energy (*R*_n_*− G*) and energy fluxes (*LE* + *H*) (Fig. 2), suggested that our ecosystem experienced an energy imbalance. The slope between *R*_n_*− G* and *LE* + *H* during the measuring period was 0.54, with an intercept of 13.87 W m^−2^ (Fig. 2A), which was obviously lower than 1. Additionally, different seasons had divergent regression slopes (Figs. 2B and 2C). During the active growing season, the regression slope could be 0.56 with an intercept of 21.68 W m^−2^ (Fig. 2B). Though the regression slope during active growing season was less than 1, it was still higher than that of the whole period (Fig. 3A) and the non-growing season, specifically (Fig. 2C). The regression slope during the non-growing season was only 0.39, with an intercept of 8.88 W m^−2^ (Fig. 2C).

The energy balance closure, as indicated by the energy balance ratio (EBR), also reflected the energy imbalance of the studied ecosystem. During the measuring period, EBR was 0.62 (Fig. 2A), which was also clearly lower than 1. However, the EBR differed among seasons (Figs. 2B and 2C). During the active growing season, the EBR measured 0.66 (Fig. 2B), which was still lower than 1 but higher than that of the whole period (Fig. 2A) and the non-growing season (Fig. 2C). The EBR during the non-growing season only measured 0.47 (Fig. 2C).

### The seasonal dynamics of ET and environmental factors

ET and its related environmental factors all exhibited obvious seasonal dynamics (Fig. 3).

Environmental factors including *T*_a_, VPD, *R*_g_, precipitation, and LAI all exhibited obvious single peak seasonal dynamics (Figs. 3A–3F). *T*_a_ ranged from −17 to 29 °C, with the highest values appearing at July, which was consistent across both years measured (Fig. 3A). VPD had higher values during the active growing season and lower values during the non-growing season; the highest VPD appeared in May (Fig. 3C). *R*_g_ ranged from 0.2 to 25 MJ m^−2^ d^−1^ and had similar dynamics to those of VPD (Fig. 3D). Precipitation also differed among seasons, with the most precipitation occurring from May to September (Fig. 3E). LAI ranged from 0.2 to 4.9 m^2^ m^−2^, with the highest values in July. RH did not show much variation across the months (Fig. 3B).

The seasonal dynamics of ET also exhibited a single-peak pattern, with the higher values appearing in the summer (Fig. 3F). However, the peak values of the seasonal dynamics of ET differed between the years measured (Fig. 3F). In 2016, ET achieved its peak (>6 mm d^−1^) in the middle of July and then decreased. In 2017, ET showed an increasing trend until the end of August, and achieved its highest value for 2017 (>7 mm d^−1^) at the end of August.

### The diurnal dynamics of ET and environmental factors

**Figure 4:**
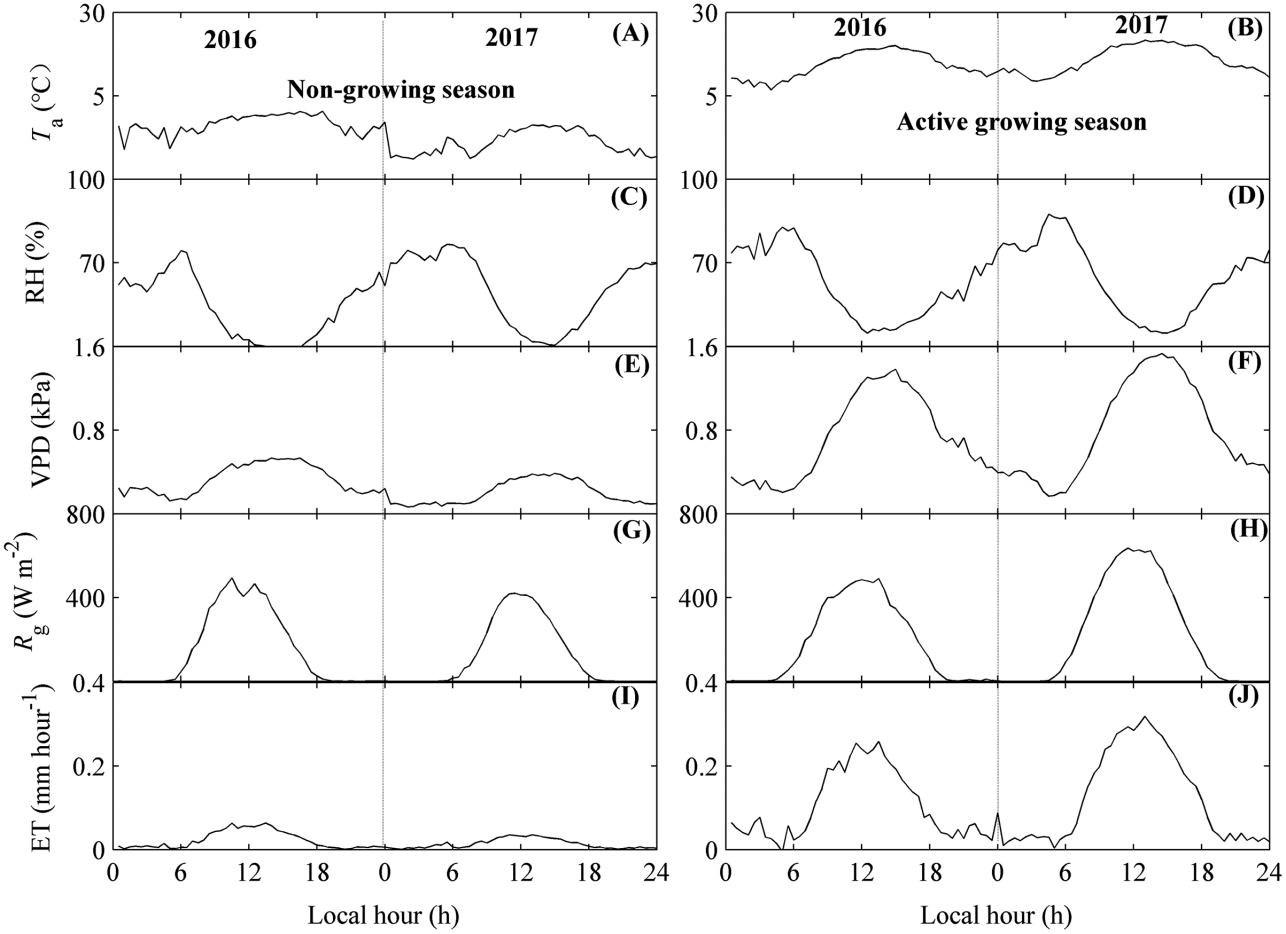
The diurnal dynamics of environmental factors and evapotranspiration (ET). Environmental factors included air temperature (*T*_a_, A and B), relative humidity (RH, C and D), vapor pressure deficit (VPD, E and F), and global radiation (*R*_g_, G and H). Diurnal variations of *T*_a_, RH, VPD, and *R*_g_ were calculated as their mean values at each 30-min period, while those of ET (I and J) were calculated by their accumulated values during each 30-min period. The diurnal dynamics were divided into the non-growing season (A, C, E, G, I) and the active growing season (B, D, F, H, J). Only directly measured and passing the quality control data were used. The fluctuations in diurnal variations of ET and environmental factors may source from the unequal number of available data in each half-hour.

ET and environmental factors all exhibited similar, obvious diurnal dynamics during the active growing season and non-growing season, but their values differed between seasons (Fig. 4).

During the day, *T*_a_ and *R*_g_ both showed single-peak patterns (Figs. 4A, 4B and 4G, 4H). These factors did not vary much from midnight to sunrise, but rapidly increased after sunrise, achieved their peak values around noon, and started to decrease at sunset. The factors were then stable until the end of the day. The time that *T*_a_ achieved its peak values (approximately 13:00 local time) was later than that of *R*_g_. However, RH exhibited a concave shape (Figs. 4C and 4D), which indicated that RH began to decrease after sunrise. VPD did not vary much during the non-growing season but showed a single peak diurnal pattern in the active growing season (Figs. 4E and 4F).

There were also similar diurnal dynamics for ET (Figs. 4I and 4J). ET showed single-peak diurnal patterns during both seasons, with the peak values occurring at 12:00 pm. However, the peak values differed between seasons. During the non-growing season, the ET peak value was no more than 0.05 mm h^−1^, while the ET peak values during the active growing season could be 0.28 mm h^−1^. These results were consisting across both years measured (Figs. 4I and 4J). Additionally, the highest diurnal peak value for ET differed between measured years, with the peak value during non-growing season in 2016 higher than that of 2017 (Fig. 4I), while the peak value during active growing season in 2016 was lower than that of 2017 (Fig. 4J).

In addition, the diurnal variations of ET and environmental factors showed some fluctuations (Fig. 4), which may source from the unequal number of available data in each half-hour.

### Drivers of ET dynamics

**Figure 5:**
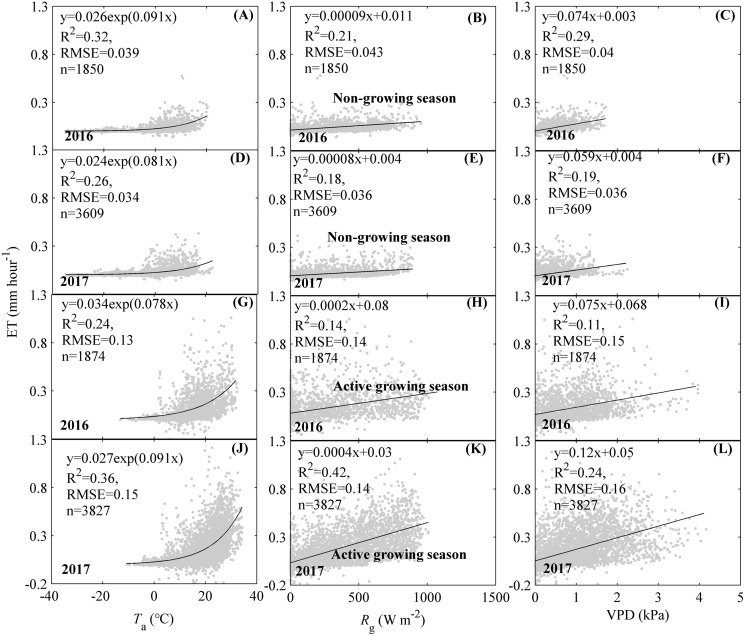
Effects of environmental factors on the diurnal variations of evapotranspiration (ET) at different seasons. Environmental factors included air temperature (*T*_a_, A, D, G, J), global radiation (*R*_g_, B, E, H, K), and vapor pressure deficit (VPD, C, F, I, L). The effects were analyzed at different seasons including the non-growing season (A–F) and the active growing season (G–L) and different years, including 2016 and 2017. Only directly measured and passing the quality control data were used.

Environmental factors including *T*_a_, *R*_g_, and VPD were found to significantly drive the single peak diurnal patterns of ET, but their effects differed among factors and seasons (Fig. 5). During the non-growing season, the increasing *T*_a_, *R*_g_, and VPD were found to significantly affect ET, while their effects differed among factors. *T*_a_ exerted the strongest effects on ET diurnal variations during the non-growing season. The equations containing *T*_a_ explained 32% and 26% of the variations for ET in 2016 and 2017, respectively (Figs. 5A and 5D). The effects of *R*_g_ and VPD on the diurnal variations of ET during the non-growing season were similar but weaker than those of *T*_a_. Therefore, *T*_a_ exerted a stronger effect on the diurnal variation of ET during the non-growing season. During the active growing season, the increasing ET was accompanied by an increase in *T*_a_, *R*_g_, and VPD, while their effects differed among factors. In 2016, *T*_a_ exerted a stronger effect on ET diurnal variations during the active growing season of 2016 (Fig. 5G), while *R*_g_ and VPD exerted weaker effects (Figs. 5H and 5I). The equation containing *T*_a_ explained 24% of the diurnal ET variations. In 2017, *R*_g_ had the strongest effect the diurnal variations of ET (Fig. 5J). The equation containing *R*_g_ explained 42% of the diurnal ET variations. *T*_a_ exerted a similar effect with *R*_g_, which were both stronger than VPD (Figs. 5J–5L). Therefore, the diurnal ET variations during the active growing season were governed by *T*_a_ and *R*_g_, respectively.

**Figure 6:**
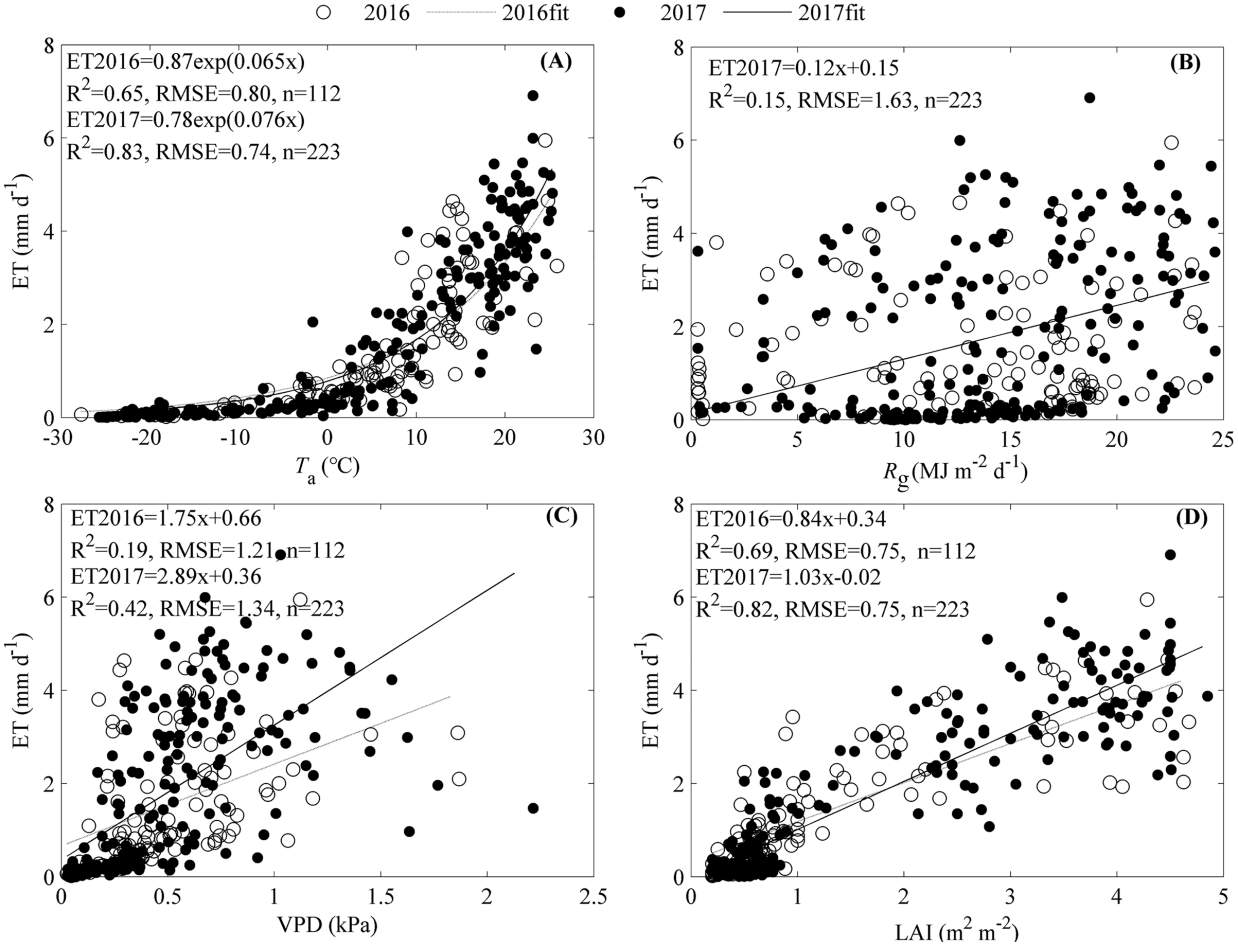
Effects of environmental factors on the seasonal variations of evapotranspiration (ET) during 2016 and 2017. Factors included air temperature (*T*a, A), global radiation (*R*_g_, B), vapor pressure deficit (VPD, C), and leaf area index (LAI, D). Only daily data during days having more than 20 (including 20) directly measured and passing the quality control data were used.

The seasonal variation of ET was primarily shaped by *T*_a_ and LAI, while *R*_g_ and VPD did not contribute much (Fig. 6). ET increased exponentially as *T*_a_ increased with an R^2^ of 0.65 and 0.83 for 2016 and 2017, respectively (Fig. 6A). *R*_g_ only significantly increased ET in 2017 with an R^2^ of 0.15 (Fig. 6B).Though the increasing VPD significantly promoted ET, the equations containing VPD explained little of the ET seasonal variations (Fig. 6C). LAI made great contributions to the seasonal variations of ET, and as it increased, ET increased linearly with an R^2^ of 0.69 for 2016 and 0.82 for 2017, respectively (Fig. 6D).

The significant correlations between ET and the environmental variables (Fig. 6) indicated that the seasonal variations of ET were shaped by multiple factors. Stepwise analysis showed that variables entering the equation describing ET seasonal variations differed between years. In 2016, only *T*_a_and LAI entered the equation describing ET seasonal variations, with an R^2^ of 0.75 and an RMSE of 0.68 mm d^−1^ (Eq. (2)). In 2017, all variables including *T*_a_, *R*_g_, and LAI entered the equation describing ET seasonal variations, with an R^2^ of 0.86 and an RMSE of 0.66 mm d^−1^ (Eq. (3)).

(2) $${\rm ET}={{0.038T_a}+{0.604}{\rm LAI}+{0.467}},\, {{\rm R^2}={0.75}},\, {{\rm RMSE}={0.68}}$$

(3) $${\rm ET}={{0.030T_a}+{0.031R_g}+{\rm 0.754LAI}-{0.092}},\, {\rm R^2=0.86}, \, {\rm RMSE}={0.66}$$

**Figure 7:**
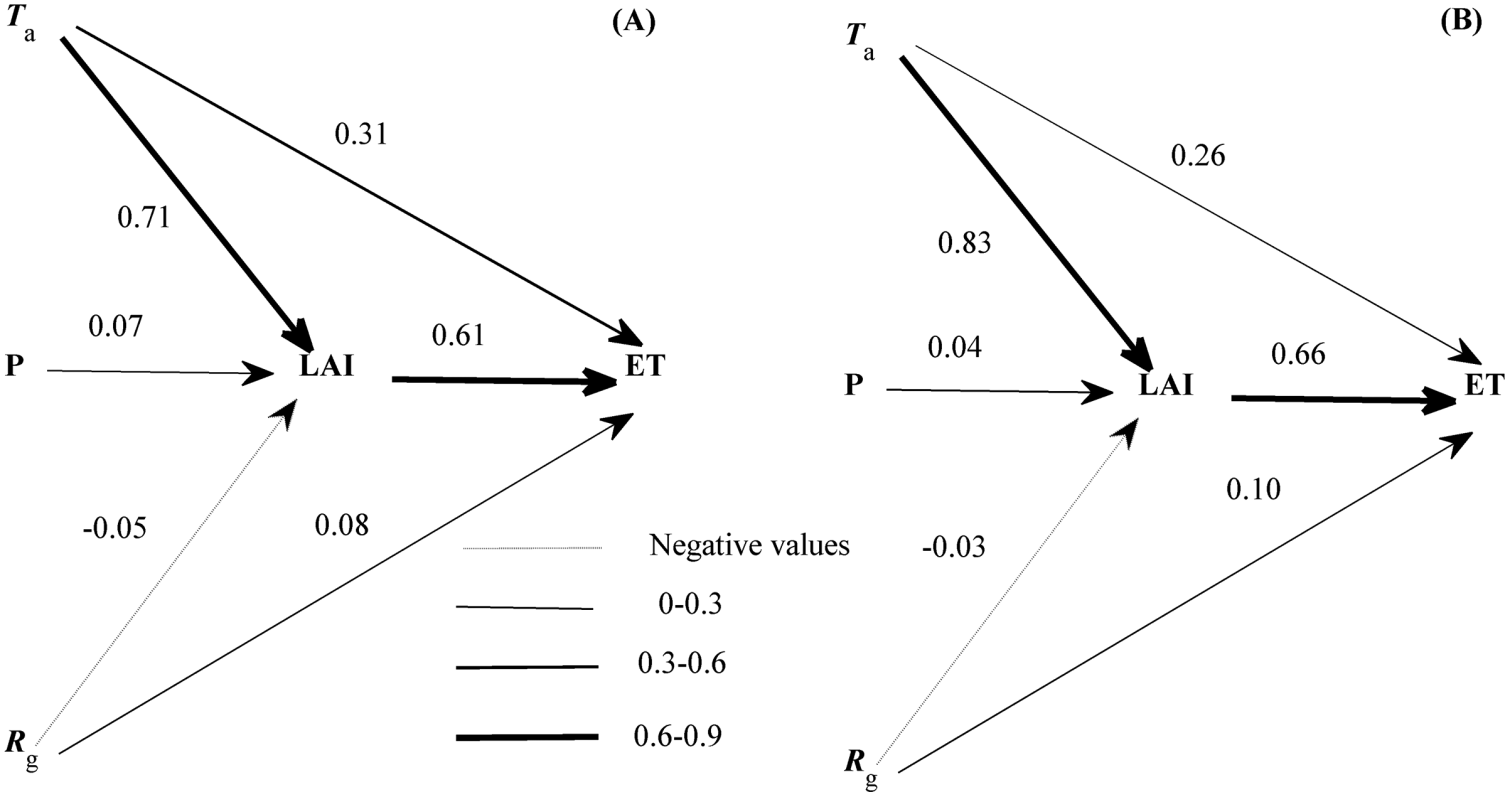
The path diagram between environmental factors and the seasonal variation of evapotranspiration (ET) at 2016 (A) and 2017 (B). The concerning variables included air temperature (*T*_a_), global radiation (*R*_g_), precipitation (P), and leaf area index (LAI). Line widths reflected the roles of each variable. Only daily data during days having more than 20 (including 20) directly measured and passing the quality control data were used.

Though many variables entered the equations describing the seasonal variations of ET (Eqs. (2) and (3)), each variable had a divergent role (Fig. 7). LAI exerted the strongest direct effect on the seasonal variation of ET. *T*_a_ and *R*_g_ also directly affected the seasonal variation of ET but showed a weaker effect than LAI. In addition, the seasonal variation of LAI was dominated by *T*_a_, and supported by *R*_g_ and precipitation. The roles of environmental factors in the seasonal variation of ET were comparable in both years (Fig. 7). LAI was shown to exert a direct effect on the seasonal dynamics of ET in our ecosystem.

### The annual amount of ET and its related variables

The mean annual ET during the measuring period was 528.26 mm year^−1^, which were measured as 501.91 ± 5.30 mm year^−1^ and 554.60 ± 11.24 mm year^−1^ for 2016 and 2017, respectively (Table 1). The uncertainty of annual ET sourced from gap-filling was less than 2%. The active growing season values from May to October were the primary contributors to ET and accounted for over 90% of its annual value (Table 1). July had the highest ET, exceeding 120 mm month^−1^ in both years, which was 1 month prior to the measurement of the highest precipitation (Table 1). However, June contributed the second highest ET in 2016 while August had the second highest ET in 2017.

**Table 1:** The monthly and annual values of evapotranspiration (ET), air temperature (*T*_a_), global radiation (*R*_g_), vapour pressure deficit (VPD), precipitation, leaf area index (LAI) during 2016 and 2017.

Year	Month	ET (mm month^−1^)	*T*_a_ (°C)	VPD (kPa)	Precipitation (mm month^−1^)	LAI (m^2^ m^−2^)
2016	1	1.28 ± 0.33	−22.12	0.04	3	0.24
	2	2.67 ± 0.17	−17.48	0.08	1.8	0.30
	3	14.45 ± 0.53	−4.51	0.24	10.9	0.43
	4	25.91 ± 0.58	4.03	0.44	27.7	0.60
	5	61.64 ± 1.73	11.95	0.73	87.5	1.57
	6	101.28 ± 2.46	16.37	0.45	140.2	3.62
	7	120.93 ± 2.52	21.01	0.65	115.5	4.26
	8	95.82 ± 2.48	18.65	0.54	70.6	4.07
	9	58.48 ± 1.96	13.38	0.33	61.9	1.72
	10	10.91 ± 0.27	−0.45	0.26	27	0.66
	11	4.82 ± 0.21	−15.09	0.07	15.7	0.43
	12	3.74 ± 0.29	−18.91	0.11	3.2	0.41
	Total/mean	501.91 ± 5.30	0.57	0.33	565.00	1.53
2017	1	0.99 ± 0.12	−20.13	0.05	2.7	0.35
	2	3.14 ± 0.10	−14.60	0.10	8.6	0.33
	3	8.56 ± 0.34	−4.83	0.23	5.8	0.46
	4	33.60 ± 1.12	4.26	0.39	63.4	0.65
	5	70.58 ± 1.06	13.11	0.88	36.7	1.89
	6	98.59 ± 2.28	16.56	0.57	123.2	3.60
	7	123.85 ± 2.53	20.99	0.76	50.9	3.71
	8	112.08 ± 3.29	19.29	0.51	94.2	3.79
	9	85.54 ± 8.64	11.74	0.39	92.9	1.57
	10	11.42 ± 0.44	1.43	0.32	16.2	0.54
	11	4.93 ± 0.13	−11.42	0.10	17.4	0.51
	12	1.32 ± 0.09	−22.35	0.10	6.5	0.25
	Total/mean	554.60 ± 11.24	1.17	0.37	518.50	1.47

**Note:**

Values of *T*_a_, VPD, and LAI were the mean values during the measuring period, while others were the total values.

The annual mean air temperature (MAT) were 0.57 and 1.17 °C for 2016 and 2017, respectively, which resulted from a hot summer from May to October and a cold winter from November to April. The annual *R*_g_ were not reported as many long data gaps during the measuring period, while VPD for 2016 and 2017 were only 0.33 and 0.37 kPa, respectively. The annual precipitation were 565 and 518.50 mm for 2016 and 2017, respectively. In addition, the annual mean LAI of 2016 and 2017 were 1.53 and 1.47 m^2^ m^−2^, respectively.

Therefore, this forest was shown to experience a high evaporate rate, which is crucial to the global water cycle.

## Discussion

The quality of our measurements was key for the accurate analysis of ET dynamics and their drivers. These were evaluated using the available data portion and energy balance closure. The available data portion may be over 43.55% of the whole year as determined by the number of observations passing data quality control, which was slightly lower than that of some ChinaFLUX ecosystems (Fu et al., 2009; Yu et al., 2008; Zhu et al., 2015a). For example, the data coverage of three forests ranged from 43% to 54% (Zhu et al., 2015a), while that of three grasslands varied from 46% to 50% (Fu et al., 2009). Our ecosystem experienced a colder winter, which affected the eddy covariance instruments negatively, resulting in a lower data quality. The relative lower data coverage of our ecosystem was acceptable. From the energy balance closure, we found that the energy balance regression slope of our ecosystem from OLSs was 0.54 and the EBC was around 0.62, which was lower than most ChinaFLUX ecosystems (Li et al., 2005) and some European forests (Moderow et al., 2009; Sanchez, Caselles & Rubio, 2010) but fell into the range of energy balance closure of FLUXNET(Stoy et al., 2013; Wilson et al., 2002). The lower EBC in our ecosystem could be attributed to three aspects. First, our ecosystem experienced a cool temperature and a multiple plant composition, which made our ecosystem have a lower EBC as the EBC would increase with the increasing mean annual air temperature (Cui & Chui, 2019) and the decreasing heterogeneity (Foken, 2008; Stoy et al., 2013; Xin et al., 2018). Second, the soil heat flux (*G*) may be underestimated, especially during the non-growing season. This was also validated by an obviously higher energy balance closure during the active growing season (Figs. 2B and 2C). *G* was the product of soil bulk density, soil heat capacity, and soil temperature variations. Our ecosystem experienced a long snow covering period, whose heat capacity was higher than the soil (Li, Jia & Lu, 2015). However, we calculated the *G* during snow covering period with a soil heat capacity at ice free seasons, which made soil heat capacity used in calculating *G* during snow covering period underestimated. Therefore, the *G* during the non-growing season was underestimated, which further decreased the EBC of our ecosystem. Third, we only considered the heat storage in the soil (*G*) but ignored other heat storages. Heat can be stored in the soil, biomass, air (Moderow et al., 2009), and biogeochemical processes (Eshonkulov et al., 2019), with nearly equal contributions among these components (Lindroth, Mölder & Lagergren, 2010). Ignoring heat storage in some components (like air and biomass) may cause the energy balance closure to be underestimated (Eshonkulov et al., 2019; Franssen et al., 2010; Lindroth, Mölder & Lagergren, 2010). We can conservatively state that the eddy covariance measurement in our ecosystem performed well after fully considering the data coverage and energy balance closure.

We analysed the dynamics of ET and their drivers based on EC measuring ET and environmental factors. Our results showed that the seasonal and diurnal variations of ET all exhibited a single peak pattern with the daily ET ranging from 0 to 7.75 mm d^−1^ and the hourly ET ranging from 0 to 0.28 mm h^−1^. We also found that diurnal dynamics of ET during the non-growing season and the active growing season were driven by *T*_a_ and *R*_g_, while seasonal ET dynamics were primarily affected by LAI. The single peak patterns of diurnal and seasonal dynamics for ET were commonly found in temperate ecosystems (Vourlitis et al., 2002; Wever, Flanagan & Carlson, 2002; Zheng et al., 2014; Zhou et al., 2010). However, our hourly and daily ET ranges differed from previous works, which may be related to a difference in climate and ecosystem types. For example, the daily ET of a Japanese temperate cypress forest ranged from 0 to 5.15 mm d^−1^ (Kosugi et al., 2007), while the daily ET of a Chinese warm temperate plantation ranged from 0 to 7.4 mm d^−1^ (Tong et al., 2017). American temperate managed forests had a daily ET ranging from 0 to 6 mm d^−1^ and hourly ET varying from 0 to 0.3 mm h^−1^ (Sun et al., 2008). The drivers also differed from other ecosystems (Tong et al., 2017; Yoshida et al., 2010), which may be related to the unique characteristics of our ecosystem. As a temperate mixed forest, our ecosystem experienced a unique climate with sufficient water supply but limited radiation. Therefore, energy supply, which could be represented by *T*_a_ or *R*_g_, was the primary factor shaping the dynamics of ET in our ecosystem. *T*_a_ showed a larger range than *R*_g_ (Figs. 5A–5F), which made *T*_a_ be the primary factor shaping ET diurnal dynamics when considering the diurnal variations of ET during the non-growing season. Given most data during active growing season of 2016 were missing (Fig. 3), the effects of environmental factors on ET diurnal variations were comparable to those during the non-growing season, which made *T*_a_ be the dominating factor shaping ET diurnal variation. However, *R*_g_ exhibited a larger range than *T*_a_ (Figs. 5G–5L) during the active growing season of 2017, which indicated that *R*_g_ governed the diurnal dynamics of ET. Though LAI was found to directly affect the seasonal dynamics of ET, LAI was the comprehensive representation of *T*_a_ and *R*_g_ (Fig. 7) and reflected the dominating role of energy supply in the seasonal dynamics of ET.

The unique climate resulted in an evaporation rate of 528.26 mm year^−1^ as ET, which was almost equal to its annual precipitation. The studied ecosystem had a lower ET than those with a warmer MAT like the Horqin grassland (Li et al., 2016). However, our ecosystem had a higher ET than other ecosystems in this region, including those experiencing a warmer MAT like the Changbaishan forest (Wu et al., 2015), Tomakomai forest (Hirano et al., 2003), and Laoshan forest (Wang et al., 2004) (Table 2), or ecosystems having a higher MAT and a lower MAP (including the Tongyu cropland, Tongyu grassland, Changling grassland) (Li et al., 2016; Liu & Feng, 2012), and ecosystems experiencing a similar MAT but a lower MAP like the Kherlenbayan-Ulaan grassland (Li et al., 2007; Liu et al., 2010). Our findings indicate that the studied ecosystem evaporated more water into the atmosphere, suggesting its importance in the global water cycle.

**Table 2:** The published annual evapotranspiration (ET) in northeast Asia.

Ecosystem	Latitude (°N)	Longitude (°E)	Altitude (m.a.s.l)	Vegetation type	MAT (°C)	MAP (mm)	ET (mm year^−1^)	Observation period	References
Changbaishan forest	42.40	128.10	736	MF	4.40	471.10	500.37	2003–2007	Wu et al. (2015), Zhang et al. (2012)
Horqin grassland	43.29	122.28	203	GRA	6.76	342.33	633.19	2008–2013	Li et al. (2016)
Tongyu cropland	44.57	122.92	184	CRO	6.73	295.92	306.33	2003–2008	Liu & Feng (2012)
Changling grassland	44.58	123.50	171	GRA	7.50	296.05	306.80	2007–2008	Dong et al. (2011)
Tongyu grassland	44.59	122.52	184	GRA	6.68	298.17	303.04	2003–2008	Liu & Feng (2012)
Laoshan forest	45.33	127.67	340	DEF	1.79	552.00	328.53	2004	Wang et al. (2004)
Tomakomai forest	42.73	141.52	140	MF	6.00	1265	367	2001–2002	Hirano et al. (2003)
Kherlenbayan-Ulaan grassland	47.21	108.74	1235	GRA	1.2	159	144.25	2003–2006	Li et al. (2007), Liu et al. (2010)
Yichun forest	48.10	129.23	420	MF	0.87	542	528.26	2016–2017	This study

## Conclusions

In this study, we analysed the dynamics of evapotranspiration (ET) in a temperate mixed forest using an eddy covariance approach. Our results showed that 43.55% eddy covariance measured data passed the data quality control checks with an energy balance ratio of 0.62. These results indicate the accuracy of the eddy covariance approach in our ecosystem. ET exhibited single-peak diurnal and seasonal patterns, with diurnal dynamics driven by air temperature (*T*_a_) and global radiation (*R*_g_) during non-growing season and active growing season, respectively, and seasonal dynamics affected by leaf area index (LAI), which all reflected the energy supply. The dynamics of ET resulted in a mean annual ET of 528.26 mm year^−1^ during 2016–2017. Therefore, the energy supply governed the dynamics of ET during all seasons and time scales in our temperate mixed forest, but variables representing the energy supply differed among seasons and time scales. The dynamics of ET show that this temperate ecosystem had an important role in global water cycles. The results of our work improve our understanding of ET dynamics in this region.

## Supplemental Information

10.7717/peerj.13549/supp-1 Supplemental Information 1 The raw 30-min evapotranspiration data and the related factors. Based on the 30-min data, daily and annual values can be obtained.

## Additional Information and Declarations

### Data Availability

The following information was supplied regarding data availability: The raw data is available in the Supplemental File.
